# Does Duration of Preoperative Sciatica Impact Surgical Outcomes in Patients with Lumbar Disc Herniation?

**DOI:** 10.1155/2014/565189

**Published:** 2014-01-28

**Authors:** Farzad Omidi-Kashani, Ebrahim Ghayem Hasankhani, Amir Reza Kachooei, Mohammad Dawood Rahimi, Reza Khanzadeh

**Affiliations:** ^1^Orthopedic Research Center, Orthopedic Department, Imam Reza Hospital, Mashhad University of Medical Sciences, Mashhad 913791-3316, Iran; ^2^Orthopedic Research Center, Mashhad University of Medical Sciences, Mashhad 913791-3316, Iran; ^3^College of Physical Education and Sport Sciences, Ferdowsi University of Mashhad, Mashhad 9177948974, Iran

## Abstract

*Background*. In lumbar disc herniation, most authors recommend nonoperative treatment for the first few weeks of presentation, but what about the upper limit of this golden period? The aim of this study is to assess the effect of preoperative sciatica duration on surgical outcome of lumbar disc herniation. *Methods*. We retrospectively evaluated 240 patients (124 males and 116 females) with a mean age of 36.4 ± 5.9 years (range 16 to 63) surgically treated due to primary stable L4-L5 disc herniation. The patients were placed into two groups: with more and less than 12-month duration of preoperative sciatalgia. Disability and pain were measured by Oswestry Disability Index (ODI) and Visual Analogue Scale (VAS). Wilcoxon test and Mann-Whitney *U* test were used for statistical analysis. *Results*. Total mean duration of preoperative sciatalgia and follow-up period were 13.3 months (range 2 to 65) and 33.7 ± 5.1 months (range 24 to 72), respectively. Comparison between the groups showed that duration of preoperative sciatalgia either less or more than 12 months did not affect the surgical outcomes significantly. *Conclusions*. More or less than 12-month duration of preoperative sciatalgia may not affect the surgical outcomes of simple lumbar disc herniation in the patients undergoing discectomy.

## 1. Introduction

Although the prevalence of lumbar disc herniation in magnetic resonance imaging (MRI) studies reaches 30%, it clinically affects only 1-2% of the people throughout their life [1, 2]. Symptomatic lumbar disc herniation is more common in male and during the fourth and fifth decades of life [3]. Natural history usually begins with a fluctuating low back pain (LBP) that eventually radiates to one of the lower extremities. The pain usually spreads below the knee.

Nonoperative treatment for four to six weeks is usually effective in 70% of the affected patients [4, 5]. In those cases with refractory complains, most authors prefer surgical discectomy to conservative treatment [6, 7]. Due to this relative benign and self-limited course of lumbar disc herniation, most authors recommend nonoperative treatment for the first few weeks of presentations, but what about the upper limit of this golden time? Some believe that too late surgical decompression is associated with worse outcomes [8–10], while others do not consider delayed surgery as a poor prognostic factor [11, 12]. The aim of this study is to assess the surgical outcome of lumbar disc herniation based on preoperative sciatalgia duration. We hypothesized that preoperative sciatalgia duration has no significant effect on surgical outcome of the patients with lumbar disc herniation.

## 2. Materials and Methods 

In this retrospective study, after local institutional review board approval (code number 89533), we analyzed our surgically treated patients due to lumbar disc herniation in our orthopedic department from September 2007 to August 2011. The inclusion criteria were a primary sciatalgia due to one-level stable lumbar disc herniation, being refractory to more than six weeks of conservative treatment, and surgical decompression without instrumentation or fusion. To avoid distortion of the results, only the patients with L4-L5 disc herniation were included. Our exclusion criteria were the cases with revision surgery, cauda equina syndrome, unstable spinal stenosis requiring fusion and/or instrumentation, significant underlying disorders (like uncontrolled diabetes mellitus, autoimmune diseases, tumors, hemorrhagic diasthesis, etc.), and a follow-up period of less than two years.

We placed our patients into two groups. The patients with preoperative sciatalgia duration less than 12 months were placed in Group A and the rest in Group B. Preoperatively, all the patients had anteroposterior and lateral standing lumbosacral radiographs, supine MRI, and in some cases electrodiagnostic studies were also performed. Disability and pain (both in lumbar region and leg) were measured by Oswestry Disability Index (ODI) and Visual Analogue Scale (VAS) on a numeric 0 to 10 scores [13–15]. These questionnaires were filled out preoperatively and at the last follow-up visits. A disability improvement of more than 10 points (20%) in ODI was considered significant clinically [16]. The patients' subjective satisfaction was also rated as excellent, good, fair, or poor.

After informing the patients about pros and cons of the surgical treatment, all the patients signed the informed consent forms and then, all operations were performed by the first author (Farzad Omidi-Kashani) with a similar surgical technique (minimal incision, unilateral exposure, laminotomy, and partial discectomy) throughout these years. As soon as the patients were able to walk and urinate, they were discharged from the hospital. In postoperative era, we also recorded the recurrence and revision rates for comparison between the groups.

*Statistical Analysis*. We used Wilcoxon test to compare postoperative improvement in pain and disability relative to preoperative status in each group. Intergroup comparison was carried out by Mann-Whitney *U* test. Statistical analysis was performed by Statistical Package for Social Sciences (SPSS) version 11.5 for Windows (SPSS Inc., Chicago, IL, USA) and *P* less than 0.05 was considered statistically significant.

## 3. Results 

After applying the inclusion and exclusion criteria, eleven patients were omitted from the study and 240 patients could fulfill our criteria. There were 116 women and 124 men with a mean age of 36.4 ± 5.9 years (range 16 to 63). The mean duration of both preoperative sciatalgia and follow-up period were 13.3 ± 4.9 months (range 2 to 65) and 33.7 ± 5.1 months (range 24 to 72), respectively.  Table 1 shows the demographic data and baseline characteristics of each group, separately.

As shown in Table 2, surgery could significantly improve leg pain, low back pain, and disability in both groups.  Table 3 compares the effectiveness of surgery in Group A with Group B regarding the improvement in pain and disability. As this table shows, duration of preoperative sciatalgia either less or more than 12 months did not affect the surgical outcomes significantly.

There were 29 cases (12.1%) with recurrent lumbar disc herniation (15 at the same level, same side; seven at the same level opposite side; seven at the different levels). From these, 12 cases (5% of the total cases) underwent revision surgery, usually with transforaminal lumbar interbody fusion and pedicular screw fixation technique. Regarding the recurrence and revision rates, there was no significant difference between the two groups. In terms of patients' satisfaction, 81% reported as excellent, 11% good, 6% fair, and 2% poor. Again, we could find no statistical difference in patients' subjective satisfaction rate between the two groups (Table 4).

## 4. Discussion

We designed a retrospective study on 240 patients who were equally placed into two groups: less and more that 12 months of preoperative sciatalgia in the patients with simple single level lumbar disc herniation. In this study, we could find no significant differences in efficacy of surgical discectomy in terms of leg VAS, lumbar VAS, and ODI improvement between the groups. Lumbar disc herniation recurrence and revision rates were also similar between the two. Our results showed that the patients were satisfied with the surgical outcomes (whether they had been operated with sciatica duration shorter or longer than 12 months).

Due to relatively benign and self-limited course of lumbar disc herniation and probable significant complications coupled with surgical discectomy, initially a minimum of six weeks of nonsurgical treatment is recommended, but after this time there are great controversies about the impact of preoperative symptom duration on the surgical outcome of lumbar disc herniation [9–12, 17, 18].

In a study conducted by Ng and Sell, on 113 consecutive patients with lumbar discectomy, the authors found a significant reverse relationship between preoperative duration of radiculopathy for more than 12 months and postoperative disability improvement [10]. In this study, surgical operations performed throughout the first 12 months of radiculopathy did not affect the outcome. Similar to our study, these authors did not find a correlation between preoperative duration of sciatalgia and postoperative VAS improvement (*P* = 0.09). In another study conducted by Nygaard et al. on 132 patients with lumbar disc herniation underwent discectomy, they found a golden time of preoperative symptoms less than eight months for obtaining better surgical outcomes and more probability of returning to work [17]. Similarly, Grøvle et al. found that duration of the sciatica more than three months was significantly associated with a longer time to return to work and worse surgical results [19].

Other proponents of this issue are Jansson et al., who in a study on 263 patients with lumbar discectomy with one-year followup, reported that smoking, short preoperative walking distance, and a long history of back pain were associated with poorer surgical outcome [18]. In the study we conducted, we did not consider smoking habit or walking ability separately and we mostly focused on ODI, leg and lumbar pain. We could not find that long duration of preoperative pain (in leg or lumbar area) is accompanied by worse surgical results.

Here, we would like to mention some studies that like us could not find an association between preoperative sciatalgia duration and postoperative surgical outcomes [11, 12]. Most of these studies are relevant to lumbar spinal stenosis (and not directly related to lumbar disc herniation) and believe that a delay of surgery does not worsen the prognosis of these patients [20, 21]. Radcliff et al. in a study the patients with spinal stenosis and degenerative spondylolisthesis evaluated the impact of preoperative symptom duration less or more than 12 months on the treatment (surgical or nonsurgical) outcomes [22]. They finally concluded that the patients with SS and symptom duration <12 months (versus >12 months) had significantly better results with surgical or nonsurgical treatment. In the patients with degenerative spondylolisthesis, preoperative symptom duration had no relationship with postoperative clinical improvement. Although in the study we conducted, the operated cases were all affected by lumbar disc herniation, we also could find no relationship between preoperative symptom duration and postoperative clinical improvement.

In conclusion, our study shows that in the patients with lumbar disc herniation undergoing discectomy, longer duration of preoperative symptoms was not associated with worse surgical outcomes. Albeit it should be noted that in our study, the number of patients with sciatica duration less than three or six months was small and consequently we could not statistically compare the impact of these durations on surgical prognosis. If we could compare the groups with these periods of time, the results might have been changed. Other shortcomings of our study included its retrospective design and its inability to evaluate other probable important factors influencing the results. It seems that the level of population culture, patient expectations, and insurance coverage may have some confounding roles. These should be proven later. It is proposed that in the future, a multicentric study in different countries throughout the world is to be carried out with a similar standard surgical and follow-up technique to avoid these confounding factors that may affect the results.

## Figures and Tables

**Table 1:** Demographic data of our treated patients.

Group	Number	Sex		Mean age ± SD^†^	Mean preoperative	Mean F/U^#^ ± SD
		Male (%)	Female (%)	(y**)	sciatica ± SD (m*)	(m)
A	113	52 (46)	61 (54)	37.4 ± 5.7	8.3 ± 5.7	32.1 ± 4.8
B	127	72 (56.7)	55 (43.3)	35.6 ± 6.5	19.2 ± 3.8	35.3 ± 5.4

^†^SD: standard deviation. **y: year. *m: month. ^#^F/U: followup.

**Table 2:** Improvement in pain and disability.

Group	Preoperative	Last visit	*z*	*P* value
Group A				
Mean VAS^#^ leg	8.16 ± 1.39	1.86 ± 2.34	4.72	0.001
Mean VAS lumbar	7.2 ± 2.09	1.8 ± 2.28	3.89	0.001
Mean ODI^†^	60.4 ± 1.67	18.46 ± 2.16	4.7	0.001
Group B				
Mean VAS leg	8.15 ± 1.7	1.5 ± 1.99	4.79	0.001
Mean VAS lumbar	7.4 ± 1.83	1.56 ± 1.69	4.71	0.001
Mean ODI	59.6 ± 1.44	16.26 ± 1.83	4.78	0.001

^#^VAS: Visual Analogue Scale. ^†^ODI: Oswestry Disability Index.

**Table 3:** Comparison between the groups in terms of pain and disability improvement.

	Mean difference between preoperative and last visit		
	VAS^#^ leg	VAS lumbar	ODI^†^
Group A	6.3 ± 2.38	5.4 ± 2.37	41.93 ± 2.88
Group B	6.5 ± 2.17	5.83 ± 2.4	43.4 ± 2.01
*Z*	0.2	0.97	0.4
*P* value	0.83	0.33	0.68

^#^VAS: Visual Analogue Scale. ^†^ODI: Oswestry Disability Index.

**Table 4:** The correlation between mean duration of preoperative sciatalgia and postoperative patients' satisfaction.

Patient satisfaction	Mean duration of preoperative sciatica (m)^†^	Group comparison	*P*
Excellent	13.6	Good	0.34
		Fair	0.62
		Poor	0.45

Good	14.6	Fair	0.61
		Poor	0.27

Fair	11.5	Poor	0.85
Poor	10.9	—	—

^†^m: month.
